# Bacterial Transformation and Processing of Diatom-Derived Organic Matter: A Case Study for *Skeletonema dohrnii*

**DOI:** 10.3389/fmicb.2022.840564

**Published:** 2022-04-28

**Authors:** Yang Liu, Xueru Wang, Jun Sun

**Affiliations:** ^1^Institute of Marine Science and Technology, Shandong University, Qingdao, China; ^2^Research Centre for Indian Ocean Ecosystem, Tianjin University of Science and Technology, Tianjin, China; ^3^College of Marine Science and Technology, China University of Geosciences, Wuhan, China; ^4^State Key Laboratory of Biogeology and Environmental Geology, China University of Geosciences, Wuhan, China

**Keywords:** organic matter (OM), dissolved OM (DOM), lysate OM (LOM), culturable epiphytic bacteria, *Skeletonema dohrnii*, dissolved organic carbon, transparent exopolymer particles

## Abstract

Bacterial transformation and processing of phytoplankton-derived organic matter are extremely important for the formation of ubiquitous organic matter (OM) in aquatic ecosystems. Heterotrophic bacteria convert OM into biomass and recycle inorganic components, contributing to the production of microbial food webs. While phytoplankton-derived organic matter is commonly studied, the transformation and processing of dissolved OM (DOM) and lysate OM (LOM) by culturable epiphytic bacteria remains poorly understood. In this study, cultivable epiphytic bacteria from the marine diatom, *Skeletonema dohrnii*, were isolated, purified, and identified. Three bacteria, *Roseobacteria* sp., *Marinobacter* sp., and *Bacillus* sp., were selected to study the transformation and processing of *S. dohrnii*-derived DOM and LOM using excitation-emission matrix (EEM) fluorescence methods, and bacterial abundance, dissolved organic carbon (DOC) concentration, and transparent exopolymer particle (TEP) content were measured. Meanwhile, the bacterial transformation of DOM and LOM was further evaluated by the fluorescence index, biological index, β/α, and humification index. The primary fluorophores, peak A (humic-like), peak C (humic-like), peak M (humic-like), peak B (protein-like), and peak T (tryptophan-like), were present in the sample. The fluorescence of DOM and LOM was dominated by protein-like signal that became increasingly humic-like over time, suggesting that more complex molecules (e.g., recalcitrant OM) are being produced. The fluorescence of DOM and LOM was dominated by a protein-like signal that became increasingly humic-like over time, suggesting that epiphytic bacteria produced more complex molecules. Results showed that the bacteria utilized LOM more rapidly than DOM. While the three bacteria transformed OM to different degrees, all were able to facilitate microbial reprocessing of OM into refractory OM.

## Introduction

Phytoplankton-derived dissolved organic matter (DOM) is the dominant form of organic matter (OM) in the aquatic environment. DOM contributes to the largest carbon stock on Earth (662 Pg C), of which more than 90% of dissolved organic carbon (DOC) is produced at a rate of 43 Tg per year (Hansell et al., 2012), and plays an important role in the global carbon cycle (Hansell et al., 2009; Thornton, 2014). The majority of DOC is modified into recalcitrant DOC and exported to the deep sea (Hansell et al., 2012). Chromophoric dissolved organic matter (CDOM) is the optically active fraction of DOM and is an essential part of the microbial process, controlling the attenuation of light and photochemical reactions, and influencing primary productivity (Blough and Siegel, 2002; Mopper et al., 2015). In comparison, lysate organic matter (LOM) from phytoplankton forms a pool of OM that is created extracellularly through metabolic excretion and adsorption (McIntyre and Guéguen, 2013). LOM can significantly protect algal cells from adverse environmental conditions (Pereira et al., 2009). Cell wall polymers of marine phytoplankton are considered one of the major components of recalcitrant dissolved organic matter (RDOM) (Tanoue et al., 1995; Benner and Kaiser, 2003). Recent studies show that carotenoid degradation products may become biological RDOM in the ocean (Arakawa et al., 2017). Fluorescent dissolved organic matter (FDOM) produced by microorganisms has also attracted wide attention due to its extended degradation time (Yamashita and Tanoue, 2008; Catalá et al., 2015). As a result, the microbial carbon pump (MCP) has been proposed to further elucidate the mechanisms by which carbon is sequestered in the ocean (Jiao et al., 2010).

Phytoplankton and bacteria have co-existed for over 200 million years (Falkowski et al., 2004), and epiphytic bacteria are essential for phytoplankton-derived DOM production (Liu et al., 2021a). Bacteria can use and transform 10–50% of the photosynthetic products of phytoplankton and together, bacteria and phytoplankton are important regulators of the structure and function of aquatic ecosystems (Seymour et al., 2017). Diatoms (Bacillariophyceae) are widespread in aquatic ecosystems and account for approximately 20% of fixed carbon in oceans (Nelson et al., 1995). Bacteria-diatom interactions typically take place in the phycosphere, a diffusive boundary layer surrounding diatom cells, or within free-floating or surface-attached cell aggregates (Amin et al., 2012). There are bacterial communities in the phycosphere that are distinct from the surrounding environment and the abundance and diversity of bacteria change during different phases of algal growth (Liu et al., 2021a). There is also specificity in the epiphytic bacteria associated with each algae species. Previous studies show that the epiphytic bacteria, *Alexandrium tamarense*, primarily consists of the genus *Roseobacter* sp. and Bacteroidetes (Jasti et al., 2005). Fandino et al. (2001) showed that free-living bacteria mainly belong to α-Proteobacteria and γ-Proteobacteria, and attached bacteria are primarily dominated by *Cytophaga–Flexibacter–Bacteroides* (CFB) during the *Lingulodinium polyedrum* dinoflagellate bloom. However, different selective pressures can impact the composition of bacterial communities in different survival environments (i.e., free-living or attached) (Grossart et al., 2005; Rooney-Varga et al., 2005). In general, epiphytic bacteria are responsible for significant uptake of monomeric carbohydrates and amino acids (Middelboe et al., 1995).

Transparent exopolymer particles (TEP) are acid-rich polysaccharides that form biologically or abiotically through condensation or exudation of extracellular polymeric substances (EPS) and can be stained by the specific dye, Alcian Blue (Passow and Alldredge, 1994; Passow, 2002). While phytoplankton are the main producers of TEP, bacteria can also release these particles (Ortega-Retuerta et al., 2010), however, the components of TEP production differ between the two. TEP produced by bacteria has a higher content of uronic acids which makes the surrounding environment more reactive with other surfaces (Bhaskar and Bhosle, 2005). As a result, bacteria are particularly important for carbon fixation by phytoplankton in aquatic ecosystems (Simon et al., 2002).

The *Roseobacter* sp. clade is one of the major groups of marine Proteobacteria, and is widely distributed in a variety of aquatic environments. Members of this clade share > 89% identity of the 16S rRNA genes (Buchan et al., 2005). *Marinobacter* sp., including halotolerant and halophilic microorganisms, is an ecologically important genus of Gammaproteobacteria found in diverse marine habitats, many species of which can degrade hydrocarbons. *Bacillus* also has the potential for degrading lignocellulosic biomass (Chen et al., 2018).

Excitation-emission matrix (EEM), also known as three-dimensional fluorescence (3D-EEM), is used commonly in fluorescence spectroscopy because it can produce a large amount of data, visual maps, and multidimensional information. EEM methods are frequently used to characterize DOM due to their remarkable sensitivity and selectivity (Coble, 1996). Protein- and humic-like fluorophores have been identified by EEM and their peak positions make them easy to discriminate.

In this study, epiphytic bacteria from the diatom *S. dohrnii* were isolated, cultured, and characterized. Given the potential importance of OM processing into biogeochemical cycles, the role and preferences of the epiphytic bacteria, *Roseobacter* sp., *Marinobacter* sp., and *Bacillus* sp., in DOM and LOM transformation by *S. dohrnii* were explored. This research builds upon laboratory-based studies lasting 90 days. The characteristics of fluorescent organic matter were examined using EEM methods and different parameters, including bacteria abundance, DOC, TEP, fluorescence index (FI), the biological index (BIX), the humification index (HIX), and *β/α* to further characterize how OM processing may differ by type of bacteria. It was hypothesized that the major bacterial taxa would correlate significantly with OM concentration and composition and would exhibit different patterns in these linkages.

## Materials and Methods

### *Skeletonema dohrnii* Cultures

Marine diatoms, *Skeletonema* spp., are widely distributed in offshore China. *Skeletonema dohrnii* was isolated from coastal seawaters around the Yellow Sea. The *S. dohrnii* was incubated at 25°C with 100 μmol photons m^–2^ s^–1^ irradiation in artificial seawater (ASW) liquid medium and the photoperiod was on a light: dark (14 h: 10 h) cycle. The conical flasks used for the ASW medium are pre-combusted (450°C, 5 h) to minimize the input of background organic carbon (Lechtenfeld et al., 2015). To regularly monitor algal growth, a 100 μL of culture solution was mounted onto a blood cell counting slide and algae cells were enumerated with a microscope (Olympus BX51, Japan).

### Isolation, Culture, and Identification of Bacterial Strains

Culturable bacteria were isolated from the degradation growth stage of *S. dohrnii* using the gradient dilution method, plated on 2216E agar plates, and cultured in transparent conical flasks (500 mL) in a shaking incubator (26°C, 160 rpm). To identify the culturable bacteria, genomic DNA was extracted using the TIANamp Bacteria DNA Kit (Tiangen-Biotech, Beijing, China). For polymerase chain reaction (PCR), amplification of the 16S rDNA V3 region, a universal bacterial primer, was used. Bar-coded fragments of the 16S rDNA were amplified using the primer 27F (5′-AGAGTTTGATCCTGGCAG-3′) and 1492R (5′-TACGGTTACCTTGTTACGACTT-3′) (Bosshard et al., 2000). A phylogenetic tree (Neighbor-Joining tree, N-J tree) was constructed using the bacterial sequences and closest related sequences from GenBank, and the genetic distances were calculated. Three isolated bacterial strains, *Roseobacter* sp., *Marinobacter* sp., and *Bacillus* sp., shared 64–100% sequence identity with the valid species from GenBank (Supplementary Figure 1).

### Flow Cytometry of Culturable Bacteria

The three culturable bacterial strains, *Roseobacter* sp., *Marinobacter* sp., and *Bacillus sp.*, were cultured in 500 mL pre-combusted (450°C, 5 h) transparent conical flasks. The abundance of bacterial cells was measured using an Accuri C6 flow cytometer (BD Biosciences, Erembodegem, Belgium), as described previously (Moens et al., 2016). The sample was stained with 0.01% SYBR Green I for 30 min in the dark at 37°C (Marie et al., 1997). As an internal standard, 1 μm fluorescent beads (Polyscience, Warrington, PA, United States) were injected into each sample. The samples were measured at a flow rate of 0.25 μL s^–1^ for 1 min.

### Measurement of Dissolved Organic Carbon and Absorption Spectroscopy

The dissolved organic carbon (DOC) was determined using a total organic carbon analyzer (TOC-3100, Germany). All samples were defrosted and acidified with phosphoric acid to a pH of 2 before being analyzed. Potassium hydrogen phthalate standards were used to quantify the DOC. Samples were gravity filtered using pre-combusted (450°C for 6 h) GF/F glass fiber filters (0.7 μm pore size, 47 mm diameter, Whatman, Maidstone, United Kingdom). While GF/F glass fiber filters do not retain as many bacterial cells as 0.2 μm polycarbonate filters, GF/F glass fiber filters can be cleaned by high-temperature combustion and can filter sufficient volumes without clogging, reducing potential sources of contamination. Therefore, the advantages of GF/F glass fiber filters prove that its application in this study is reasonable.

### Transparent Exopolymer Particle Measurements

Lysate OM samples (100 mL) were filtered through duplicate 0.4 μm polycarbonate membranes (Millipore, Billerica, MA, United States) at low and constant pressure (<100 mm Hg) and particles retained on the filters were stained for < 5 s with 0.5 mL Alcian Blue solution (0.02% w/w at pH 2.5, Alcian Blue 8GX, Sigma) that had been prefiltered through 0.2 μm polycarbonate membranes (Millipore, United States) to fully remove reaggregated dye particles. Stained filters were rinsed twice with distilled water to remove excess dye that failed to bond with substrates. Alcian Blue-stained particles were extracted from filters by soaking in 6 ml of 80% H_2_SO_4_ for 2 h with gentle agitation at least three times during this period. The extracted TEP was then measured using spectrophotometry (referring to Passow and Alldredge, 1995) with a maximum absorption of 787 nm. Calibrations by the Gum Xanthan (GX, Sigma) were carried out for the recorded TEP absorbance values and the final results were expressed in micrograms of GX equivalents per liter (μg Xeq L^–1^) using the following equation:


$${\text{C}}_{\text{TEP}}=\left(E_{787}-B_{787}\right)\times {\left(V_{\text{f}}\right)}^{-1}\times f_{\text{x}}$$

where E_787_ is the sample absorption, B_787_ is the blank (distilled water) absorption, V_*f*_ is the filtered volume in liters, and f_*x*_ is the calibration factor (Bittar et al., 2018).

### Excitation-Emission Matrix Fluorescence Spectroscopy

Three dimensional (3D)-EEM measurements were made using a fluorescence spectrophotometer (Hitachi F-7100, Tokyo, Japan). The voltage of the photomultiplier tube was set to 700 V. Fluorescence spectra detected subsequent scanning of excitation (Ex) from 200 to 450 nm and emission (Em) from 250 to 550 nm. Ex and Em slits were maintained at 5 nm and the scanning speed was set at 12000 nm min^–1^. Instrument corrections were performed based on the procedure recommended by the Hitachi F-7100 instruction manual. To eliminate most of the Raman scatter, each DOM spectrum was subjected to blank subtraction using ultrapure water (Milli-Q). The correction was followed by Raman calibration as described previously (Lawaetz and Stedmon, 2009). A number of fluorescent peaks were used in this study, namely peak A, peak C, peak M, peak B and peak T. The components of these peaks include: humic-like (peaks A, C, and M) and protein-like (peak T). All peak characteristics mentioned are referenced in Table 1. Fluorescence intensity arbitrary units (a.u.) were utilized to analyze all data. Four indexes [including FI (McKnight et al., 2001), BIX (Huguet et al., 2009), *β/α* (Parlanti et al., 2000), and HIX (Ohno, 2002)] were calculated using the formula below:

**TABLE 1:** Central regions of EEM fluorescence attributed to different sources of organic matter compared with previous studies.

Traditional peak	Ex/Em	Description	Probable origin	Comparison with previous studies
Peak A	250(325)/425	Humic-like	Terrestrial	Humic-like C1: 320 (250)/422 (Yamashita et al., 2011) Humic-like C4: 325 (250)/416 (Stedmon et al., 2003)
Peak C	320–360/420–460	Humic-like	Terrestrial/Autochthonous	Humic-like P8: < 260 (355)/434 (Murphy et al., 2008)
Peak M	290–310/370–420	Humic-like	Microbial processing of organic matter	Humic-like P1: 310/414 (Murphy et al., 2008)
Peak B	225(275)/305	Protein-like	Autochthonous tyrosine-like fluorescence	Tyrosine-like Peak B: 225(275)/305 (Coble et al., 1998; Para et al., 2010)
Peak T	225(275)/330–340	Protein-like Tryptophan-like	Autochthonous/Amino acids, free or bound in proteins	Tryptophan-like, protein-like Peak T: 225(275)/340 (Coble, 1996; Coble et al., 1998)
Peak T1	275/330–340	Tryptophan-like	Autochthonous/Amino acids, free or bound in proteins	Tryptophan-like, protein-like Peak T: 275/340 (Coble, 1996; Stedmon et al., 2003)
Peak T2	225–230/330–340	Tryptophan-like	Autochthonous/Amino acids, free or bound in proteins	Tryptophan-like, protein-like Peak T: 225/340 (Coble, 1996; Stedmon et al., 2003)


(1) $$\text{FI}=\left(I_{\text{Ex}=370},I_{\text{Em}=470}\right)\,/\,\left(I_{\text{Ex}=370},I_{\text{Em}=520}\right)$$

(2) $$\text{BIX}=\left(I_{\text{Ex}=310},\,I_{\text{Em}=380}\right)\,/\,\left(I_{\text{Ex}=310},\,I_{\text{Em}=430}\right)$$

(3) $$\beta /\alpha =\left(I_{\text{Ex}=310},I_{\text{Em}=380}\right)\,/\,\left(I_{\text{Ex}=310},\sum I_{\text{Em}=420\,\text{∼}\,435}\right)$$

$$\text{HIX}=\left(I_{\text{Ex}=254},\sum I_{\text{Em}=435\,\text{∼}\,480}\right)$$

(4) $$/\,\left(I_{\text{Ex}=254},\sum I_{\text{Em}=300\,\text{∼}\,345}+\sum I_{\text{Em}=435\,\text{∼}\,480}\right)$$

where *I* is the fluorescence intensity at each wavelength. Ex and Em are represented as excitation wavelength and emission wavelength, respectively.

The FI greater than 1.8 indicate fluorescence component of OM was mainly produced by microorganisms (Loginova et al., 2016). The BIX can be used as indicators of DOM traceability in aquatic ecosystems (Huguet et al., 2009). The *β/α* (a ratio of two known fluorescing components, where β represents more recently derived DOM and α represents highly decomposed DOM) (Parlanti et al., 2000). The HIX is a fluorescence index of organic matter degradation (Ohno, 2002).

### Experimental Set-Up

Experiments were conducted in transparent conical flasks that were pre-acid washed and rinsed with ultrapure water. *S. dohrnii* cells were cultivated in ASW medium, and after reaching the degradation growth phase, in which the algal concentration was approximately (3.84 ± 0.01) × 10^7^ cells L^–1^, microalgae liquid was filtered through a 0.2 μm polycarbonate membrane (Millipore, United States) to remove the particles. To prevent carbon contamination, the filter system was pre-cleaned with Milli-Q water, and the filtrate was regarded as the DOM fraction. To isolate the LOM fraction, algal samples were centrifuged at 5000 rpm for 5 min. The supernatant was removed, resuspended with 0.05% NaCl solution, and heated at 60°C for 30 min. The extracted solutions were then centrifuged at 7000 rpm for 20 min and the supernatant was regarded as the LOM fraction (Xu and Jiang, 2013). LOM samples were filtered through a 0.2 μm polycarbonate membrane (Millipore, United States) to prevent the introduction of particulate matter. We collected, concentrated, centrifuged and washed the bacterial cells to prevent the effect of the substrate on the growth of the bacteria. To evaluate the ability of epiphytic bacteria derived from *S. dohrnii* to transform DOM and LOM, the following treatment groups were established: (1) *Roseobacter* sp. grown in DOM (*Roseobacter* sp. + DOM), (2) *Marinobacter* sp. grown in DOM (*Marinobacter* sp. + DOM), (3) *Bacillus* sp. grown in DOM (*Bacillus* sp. + DOM), (4) *Roseobacter* sp. grown in LOM (*Roseobacter* sp. + LOM), (5) *Marinobacter* sp. grown in LOM (*Marinobacter* sp. + LOM), and (6) *Bacillus* sp. grown in LOM (*Bacillus* sp. + LOM). Two blanks, (1) DOM filtrate (DOM Blank), and (2) LOM filtrate (LOM Blank), were also used. A total of eighteen 1.8 L borosilicate bottles per treatment were inoculated with an initial abundance of 7.25–7.27 × 10^5^ bacteria cells mL^–1^, and the room temperature was maintained at 26°C. Three independent replicates were analyzed per experimental group (i.e., 24 conical flasks for 6 treatment group and 2 control group). In brief, all experiments were carried out in triplicate and under dark conditions.

### Statistical Analysis

In this study, data were checked for normality and homogeneity of variance before analysis. A one-way analysis of variance (ANOVA) and *t*-tests via SPSS software (version 23.0) were used to analyze the data. If an ANOVA was significant, differences between DOM and LOM treatment groups (excluding DOM and LOM blank groups) were compared with a Tukey’s *post hoc* test. The *p* < 0.05 was considered statistically significant. The correlation analysis and DOM associated parameters were obtained using the “ggplot2” and “corrplot” package in RStudio software. Pearson correlation coefficients were used to evaluate the relationships between fluorescence index and fluorescence peak intensity. The measured values were expressed as the mean ± standard deviation (SD).

## Results

### Bacterial Abundance, Dissolved Organic Carbon, and Transparent Exopolymer Particle Concentrations

**FIGURE 1:**
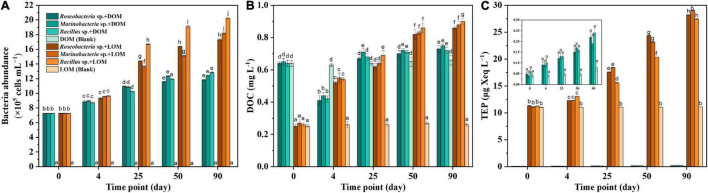
Bacteria abundance **(A)**, DOC (dissolved organic carbon, **B**) and TEP (transparent exopolymer particles, **C**) for the different treatment groups and associated blanks. Error bars represent the standard error for triplicates cultures. Comparison between all DOM and LOM treatments for all experimental days. Different lowercase letters represented significant difference (*p* < 0.05).

Growth of the three culturable bacterial strains, *Roseobacter* sp., *Marinobacter* sp., and *Bacillus* sp., in different treatment groups, is shown in Figure 1A. In all groups, the initial abundance of bacteria was around (7.26 ± 0.01) × 10^5^ cells mL^–1^, and this increased over the course of the 90 days incubation period. While *Bacillus* sp. + DOM achieved the highest abundance of up to (12.86 ± 0.08) × 10^5^ cells mL^–1^, *Marinobacter* sp. + DOM had a moderate abundance of (12.42 ± 0.03) × 10^5^ cells mL^–1^ and the abundance of *Roseobacter* sp. + DOM slowly increased to (11.84 ± 0.09) × 10^5^ cells mL^–1^. In contrast, bacterial abundance increased markedly in all the LOM groups, especially in the *Bacillus* sp. + LOM group, which increased to (20.25 ± 0.11) × 10^5^ cells mL^–1^. The significant increase in bacterial abundance seen in all treatment groups was likely caused by the uptake of organic matter.

As shown in Figure 1, the initial DOC concentration was 0.64 ± 0.01 mg L^–1^ for the DOM treatment groups and decreased over the four days of culture. The DOC concentration increased slightly on the remaining days but this difference was not significant. In contrast, the DOC concentration of the LOM treatment groups maintained an increasing trend. The largest DOC increase occurred in the *Bacillus* sp. + LOM group, which rose by 0.64 mg L^–1^ DOC over the 90-day period.

Figure 1C shows the TEP trend in the DOM and LOM treatment groups. It was visually apparent that while TEP concentrations increased in the DOM groups, they remained low. For example, *Marinobacter* sp. + DOM only increased from 0.04 ± 0.01 to 0.19 ± 0.03 μg Xeq L^–1^. TEP concentrations in the LOM treatment groups increased significantly over time.

### Fluorescence Characteristics

**FIGURE 2:**
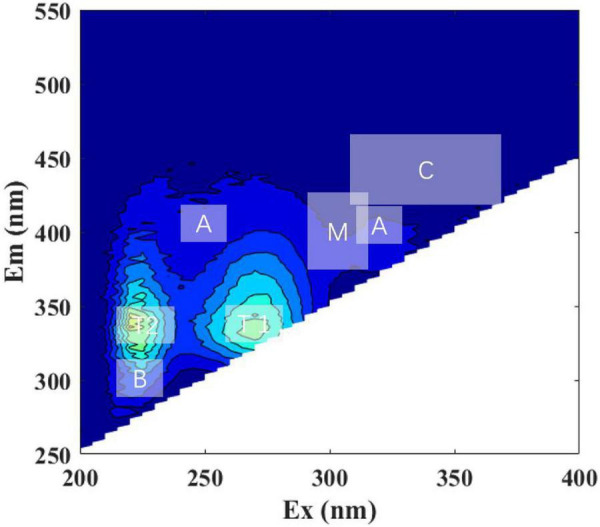
Representative excitation-emission matrices (EEMs) of *Roseobacteria* sp. + DOM treatment at initial time point (day 0). Major peak regions of EEMs are indicated in the picture. The ‘A,’ ‘C,’ and ‘M’ stands for humic-like; ‘B’ and ‘T’ stands for protein-like; ‘T1’ and ‘T2’ stands for tryptophan-like.

Fluorescence patterns were similar between all the DOM and LOM treatment groups, with peak T (including T1 and T2, protein-like) being the most prominent (Figure 2, Supplementary Figures 2, S3, and Table 1). The peak T (T1 and T2) of the *S. dohrnii*-derived DOM was more pronounced until the overall peak pattern shifted to longer wavelengths. *Roseobacter* sp. + DOM maintained the two-peak pattern during the first 25 days (Supplementary Figures 2A–C). On day 50, a weaker peak M (Ex/Em, 335/400, humic-like) appeared (Supplementary Figure 2D). This phenomenon was also seen in the *Marinobacter* sp. + DOM and *Bacillus* sp. + DOM cultures (Supplementary Figures 2I,N), but was not significant. *Marinobacter sp.* + DOM showed a clear peak B (Ex/Em, 225/300, protein-like) on day four (Supplementary Figure 2G), after which the fluorescence characteristics weakened and disappeared. The overall fluorescence characteristics of *Bacillus* sp. + DOM had fluorescence characteristics that were similar to *Roseobacter* sp. + DOM, with peak M (humic-like) being relatively weak. Higher fluorescence intensities generally occurred on days 4 and 25 for all DOM treatment groups.

The overall peak pattern of LOM was similar to that of DOM (Supplementary Figures 2, 3), but these peaks were broader and less distinct. In all LOM treatment groups, there was an initial low-intensity fluorescence (a.u. < 800) in the region of peak T2 (Ex/Em, 275/340) (Supplementary Figures 3A,F,K). Peak T1 was relatively weak compared to the DOM treatment groups. On day four, the LOM treatment groups essentially formed a three-peak pattern (Supplementary Figure 3B,G,I), and unstructured fluorescence with slightly enhanced fluorescence appeared around peaks T1 and T2. The maximum fluorescence intensity gradually decreased over time. On day 90, the fluorophore changes in *Marinobacter* sp. + DOM were not as obvious as those seen in *Roseobacter* sp. + LOM and *Bacillus* sp. + LOM (Supplementary Figures 3C,J,O).

### Fluorescence Indices and Peaks

**FIGURE 3:**
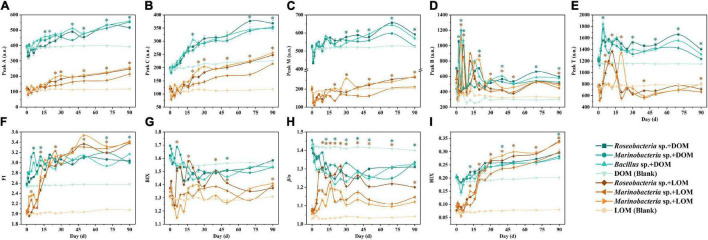
Changes in fluorescence peaks **(A–E)** and indices **(F–I)** of three culturable epiphytic bacteria, *Roseobacteria* sp., *Marinobacter* sp., and *Bacillus* sp. of *S. dohrnii* in the different OM treatments over the 90-day time period. Error bars represent the standard error for triplicates cultures. Note error bars are smaller than the symbol sizes in some cases. Tukey’s *post hoc* test were conducted between the DOM and LOM treatments, excluding the blank groups. Asterisk (*) represents statistical significance at the *p* < 0.05. Green and orange asterisk indicate statistical analysis for each experimental day for the DOM and LOM groups, respectively.

Peaks A and C (humic-like) had similar fluorescence intensities in all treatment groups, which increased over time (Figures 3A,B). Peak M (humic-like) declined for the first two to three days and then increased slowly in all treatment groups (Figure 3C). For peak B, within-group samples were similar for the DOM and LOM treatment groups, except for *Bacillus* sp. + LOM which had the highest value at day 20 and then decreased (Figure 3D). For peak T, DOM treatment groups had the highest value on day four, while LOM treatment groups peaked on different days (Figure 3E).

The fluorescence index was also used to indicate changes in fluorescence characteristics. Changes in the fluorescence indices, FI, BIX, β/α, and HIX, are shown in Figure 3. In general, LOM values were lower than DOM values across all fluorescent indices, with similar trends for FI and HIX (Figures 3F,I), and for BIX and β/α (Figures 3G,H). Compared to the DOM treatment groups, the LOM treatment groups showed a clear increase in FI and HIX. In addition, BIX and *β/α* decreased over time in the DOM treatment groups (Figure 3G).

## Discussion

Most previous studies have investigated the dynamics of phytoplankton and bacterial CDOM with carbon and nutrient additions (Romera-Castillo et al., 2010; Goto et al., 2017, 2020). It has also been shown that phytoplankton produce DOM with different characteristics and fluorescence intensities under different growth conditions (Liu et al., 2021a,b). To better investigate the transformation of OM by culturable marine bacteria, diatom-derived DOM and LOM were used as the sole carbon sources.

In this study, culturable epiphytic bacteria were isolated from *S. dohrnii*, and identified. The 90-day transformation of *S. dohrnii* DOM and LOM by three bacterial strains, *Roseobacter* sp., *Marinobacter* sp., and *Bacillus* sp., was assessed. *S. dohrnii*-derived DOM was collected during degradation because it had multiple fluorescence characteristics and high fluorescence intensity. To ensure consistency of the experimental materials, *S. dohrnii*-derived LOM was also extracted during degradation. It was helpful to observe the transformation of DOM and LOM by epiphytic bacteria. Previously published data along with results from this study demonstrate that the characteristics of OM produced by diatoms are relatively similar (Liu et al., 2021a). However, while the fluorescence characteristics of *S. dohrnii*-derived DOM and LOM were similar, the fluorescence intensity of LOM was generally low (≤1200 a.u.).

### Spectral Properties of *Skeletonema dohrnii*-Derived Dissolved Organic Matter and Lysate Organic Matter

All treatment groups produced a two- or three-peak patter dominated by discrete *S. dohrnii*-derived DOM and LOM fluorophores, indicating that different compounds, such as those resembling tryptophan, are present in algae CDOM. In addition, not all fluorophores were created from contamination during incubation or by the background ASW medium. Several studies have shown that protein peaks (e.g., tryptophan-like) are dominant during phytoplankton growth (Stedmon and Markager, 2005; Jørgensen et al., 2011; Liu et al., 2021a,b). In addition, peaks A and C (*e.g.*, flavins and phenols) are thought to be humic substances from the earth (Wünsch et al., 2015). As a result, the experiments were conducted with an inorganic medium synthesized from artificial seawater, which lacks substances of terrestrial origin. Thus, the observed changes in unstructured fluorescence with slightly enhanced fluorescence, the formation of peaks A and C, and the subsequent red-shift were due to bacterial-transformed DOM and LOM rather than terrestrially derived fluorescence. DOM and LOM fluorescence had similar peak regions and DOM usually had a broader emission wavelength. It is likely that once algae are degraded, the algal-derived substances are released into the surrounding aquatic environment. In previous studies, DOM produced in phytoplankton and bacterial cultures showed similar fluorescence patterns as those presented here (Kinsey et al., 2018). Fukuzaki et al. (2014) also studied the DOM spectra of algal cultures (e.g., axenic diatoms, dinoflagellates, and chlorophytes) and found that even when DOM fluorophores are different, the DOM patterns produced are generally similar. In the current study, the fluorescence of peaks A, C, and T increased, while peak B increased significantly in a short period of time and then decreased to near its initial value. This suggests that epiphytic bacteria utilize DOM and LOM over a short period which leads to a brief increase in bacterial abundance before the OM is transformed into recalcitrant OM and the epiphytic bacteria are gradually accumulated. The incubations contained the protein-like fluorophore (e.g., tryptophan-like components), which increased by the fourth day likely because the production of bacteria exceeded consumption (Goto et al., 2017, 2020). Importantly, these changes occurred in all treatment groups. Specifically, there was a significant increase in peak B and T fluorescence intensity in the short term, which then decreased. This suggests that large polymers were still released but that some of these exopolymers were degraded into small peptides (Goto et al., 2017). The decrease in the tryptophan-like fluorophore was predominant, but stayed relatively stable for the remaining time, suggesting that small peptides produced by the degradation of large exopolymers correlated with reutilization of tryptophan-like fluorophores by the bacteria.

Previous studies of natural microbial community cultures have shown that a wide range of heterotrophic bacteria primarily produce humic-like substances during growth (Shimotori et al., 2009; Arai et al., 2018). It is noteworthy that these humus-like substances are recalcitrant during deep ocean circulation and only a small fraction of fluorescent dissolved organic matter are biodegradable (Catalá et al., 2015). In addition, the generally low fluorescence in the open ocean suggests that the degradation experiments in this study were conducted over a longer time period than those performed in other studies (Gruber et al., 2006; Fukuzaki et al., 2014). One study demonstrated that a single bacterial species cannot completely degrade large molecular weight OM compounds, implying that multiple species are required for degradation to occur (Horemans et al., 2013). The utilization and transformation of OM by a single bacterium takes considerably longer. The current study shows that the transformation and utilization of DOM by a single strain of bacteria requires more time than the transformation of LOM (Liu et al., 2022).

The FI provided a clear insight into the process of OM-related substance changes. The values were all greater than 1.8, indicating that the fluorescence component of OM was mainly produced by microorganisms (Loginova et al., 2016). The FI values of the LOM treatments rose significantly, surpassing the DOM treatments by day 40. The ratio of two known fluorescing components (β/α, where β and α represent more recently derived OM and highly decomposed OM, respectively) and BIX, where higher values indicate greater degradation of DOM. The significant changes in β/α and BIX values over a short period of time indicate that endogenous carbon products are most likely produced through bacterial processing of DOM and LOM. The higher values of HIX indicating higher molecular weight aromatic compounds (i.e., HIX is directly proportional to the humic content of DOM) (Huguet et al., 2009). The degree of humification gradually intensified over time in all treatment groups, especially the LOM groups (*e.g.*, *Bacillus* sp. + LOM). These findings indicated that the alteration of the OM pool by bacteria leads to a tendency for the fluorescent component to become humified. Taken together, these results indicate that epiphytic bacteria used in this study have a strong ability to transform *S. dohrnii*-derived LOM, while only weakly transforming *S. dohrnii*-derived DOM.

### Organic Matter Transformation

Transparent exopolymer particle is a class of exopolymers rich in acidic polysaccharides that form biotically or abiotically by exudation or coagulation of exopolymeric precursors (i.e., exopolymeric substances) (Alldredge et al., 1993). TEP is widely distributed in diatoms, coccolithophores, and cyanobacteria (Van Oostende et al., 2013; Chen and Thornton, 2015; Pannard et al., 2016). The current study compared epiphytic bacteria TEP production needed to utilize *S. dohrnii*-derived DOM and LOM. The DOM-treated groups showed a small amount of TEP production that increased slowly over time, indicating that epiphytic bacteria can produce and release TEP, as shown previously (Bhaskar and Bhosle, 2005). In contrast, the TEP concentration increased significantly in the LOM-treated groups indicating that the epiphytic bacteria were able to better utilize LOM and synthesize their own biomass.

In the present study, the significant positive correlation (*p* < 0.05) between bacterial abundance and different carbon pools (i.e., DOM and LOM) suggested a strong link between bacterial growth and activity, with epiphytic bacteria actively transforming organic compounds. The DOC concentration showed a distinct increase (net increases of 0.11–0.55 mg L^–1^), suggesting that OM components were transformed by bacteria to produce new DOC (i.e., production was higher than consumption). This is similar to DOC concentrations (0.41–0.96 mg L^–1^) observed in the natural environment (Hansell et al., 2009). The OM can be used as a substrate for remineralization of carbon by heterotrophic microorganisms. Microorganisms use enzymes to catalyze OM into smaller compounds that can be transported to the environment across bacterial cell membranes (Arnosti, 2011). Organic carbon is then incorporated into the biomass or excreted as DOC in the form of metabolic products (Arnosti, 2011). This provides a new perspective for observing the transformation process of diatoms-derived DOM and LOM by epiphytic bacteria.

### Correlation Between Fluorescence Indices and Peak Parameters

**FIGURE 4:**
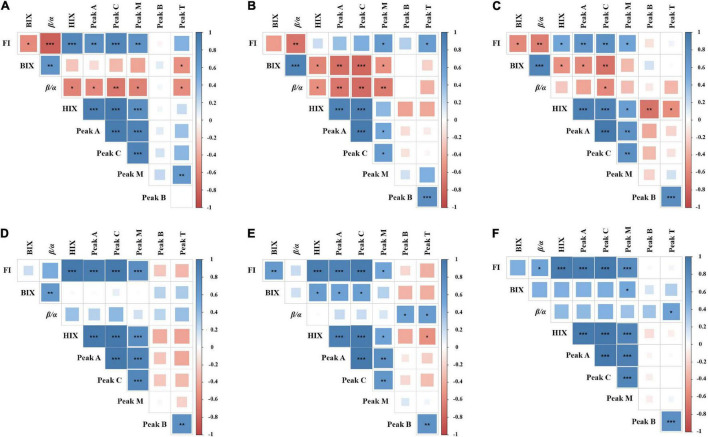
Correlation matrix among different fluorescence peaks and indices parameters. **(A–C)** Represent *Roseobacter* sp. + DOM, *Marinobacter* sp. + DOM, and *Bacillus* sp. + DOM, respectively. **(D–F)** Represent *Roseobacter* sp. + LOM, *Marinobacter* sp. + LOM, and *Bacillus* sp. + LOM, respectively. The color and size of the square circle indicate the Pearson’s correlation coefficient. *, **, and *** indicate significance levels at *p* < 0.05, *p* < 0.01, and *p* < 0.001, respectively. The blue color indicates a positive correlation and the red color indicates a negative correlation.

By analyzing the relevant parameters of fluorescence indices and peaks, it was found that most parameters were correlated significantly, either positively or negatively (Figure 4). However, there were differences between each treatment group. Indeed, significant correlations were found between HIX and other parameters. In the DOM treatment groups (Figures 4A–C), the HIX was positively correlated with the FI, peak A, peak C, and peak M, but negatively correlated with the peak B, peak T, β/α, and BIX. Since HIX is a fluorescence index measuring the degradation of organic matter (aromatic compounds), with higher values indicating higher molecular weight, the results demonstrate that DOM is gradually converted to recalcitrant high molecular weight compounds by epiphytic bacteria. In the LOM treatment groups (Figures 4A–C), peak B was negatively correlated with most parameters (e.g., FI, HIX, peaks A, C, and M) and showed different correlations in each DOM treatment group. This observation suggests that epiphytic bacteria have different transforming abilities for DOM and LOM.

## Conclusion

This study demonstrates that epiphytic bacteria are involved in the formation of unstructured “humus-like” fluorescence. In short, epiphytic bacteria are essential for the transformation and processing of diatom-derived OM. Previous studies have observed red-shift of CDOM peaks and these have been described as the result of microbial degradation (Burdige et al., 2004; Kinsey et al., 2018). This supports the conclusion that the altered fluorescence peaks and indices observed in this study are due to bacterial processing of DOM and LOM, and reprocessing into more complex molecules like RDOM (Lechtenfeld et al., 2015). The presence of molecules that are harder to degrade can facilitate charge transfer interactions between electron acceptors and donors. Indeed, the interaction between heterotrophic bacteria and OM is also an important source of deep-sea RDOM.

## Data Availability Statement

The original contributions presented in the study are included in the article/Supplementary Material, further inquiries can be directed to the corresponding author/s.

## Author Contributions

JS and YL: conceptualization. JS: methodology, resources, supervision, project administration, and funding acquisition. YL: writing—original draft preparation and visualization. YL, XW, and JS: writing—review and editing. All authors: read and agreed to the published version of the manuscript.

## Conflict of Interest

The authors declare that the research was conducted in the absence of any commercial or financial relationships that could be construed as a potential conflict of interest.

## Publisher’s Note

All claims expressed in this article are solely those of the authors and do not necessarily represent those of their affiliated organizations, or those of the publisher, the editors and the reviewers. Any product that may be evaluated in this article, or claim that may be made by its manufacturer, is not guaranteed or endorsed by the publisher.
