# Supplementary material for: Bacterial Transformation and Processing of Diatom-Derived Organic Matter: A Case Study for Skeletonema dohrnii
Source: Front Microbiol. 2022 Apr 28;13:840564. doi: 10.3389/fmicb.2022.840564 (PMC9096949; doi:10.3389/fmicb.2022.840564)
Supplement: Supplementary file 1 [file Table_1.DOC]

**Journal: *Frontiers in Microbiology***

Supplementary information for

**Bacterial transformation and processing for diatom-derived organic matter: a case study for *Skeletonema dohrnii***

Yang Liu^1,2^, Xueru Wang^2^, Jun Sun^2,3,4^*

^1^ Institute of Marine Science and Technology, Shandong University, Qingdao, Shandong 266237, China

^2^ Research Centre for Indian Ocean Ecosystem, Tianjin University of Science and Technology, Tianjin 300457, China

^3^ College of Marine Science and Technology, China University of Geosciences (Wuhan), Wuhan, Hubei 430074, China

^4^ State Key Laboratory of Biogeology and Environmental Geology, China University of Geosciences (Wuhan), Wuhan Hubei 430074, China

**Contents of this file:**

- **Figures: 3**


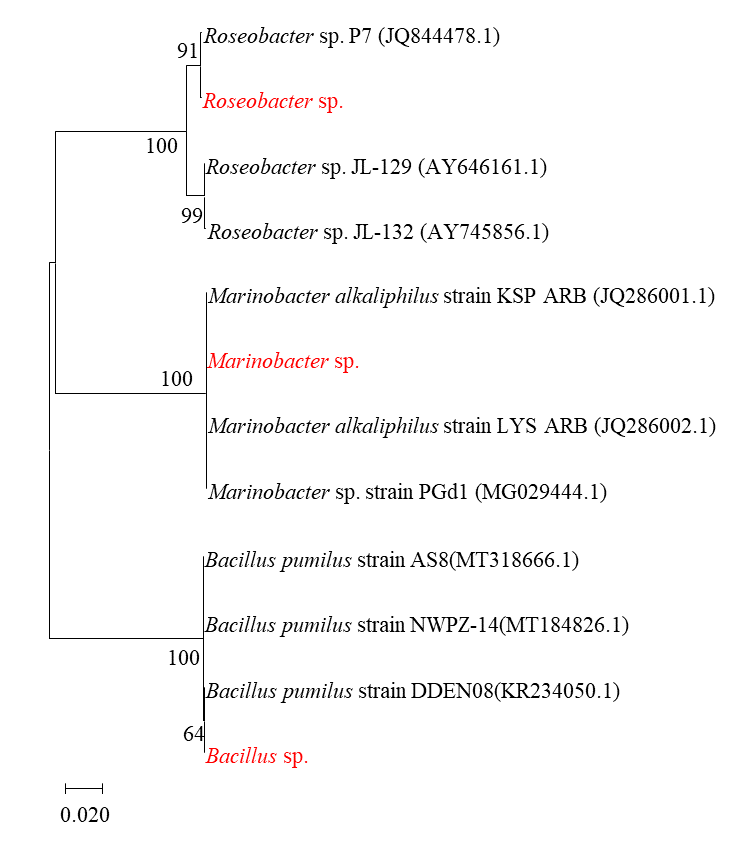


**Figure S1 The phylogenetic tree of the isolated culturable epiphytic bacteria based on NJ analysis of the 16S rRNA gene.**


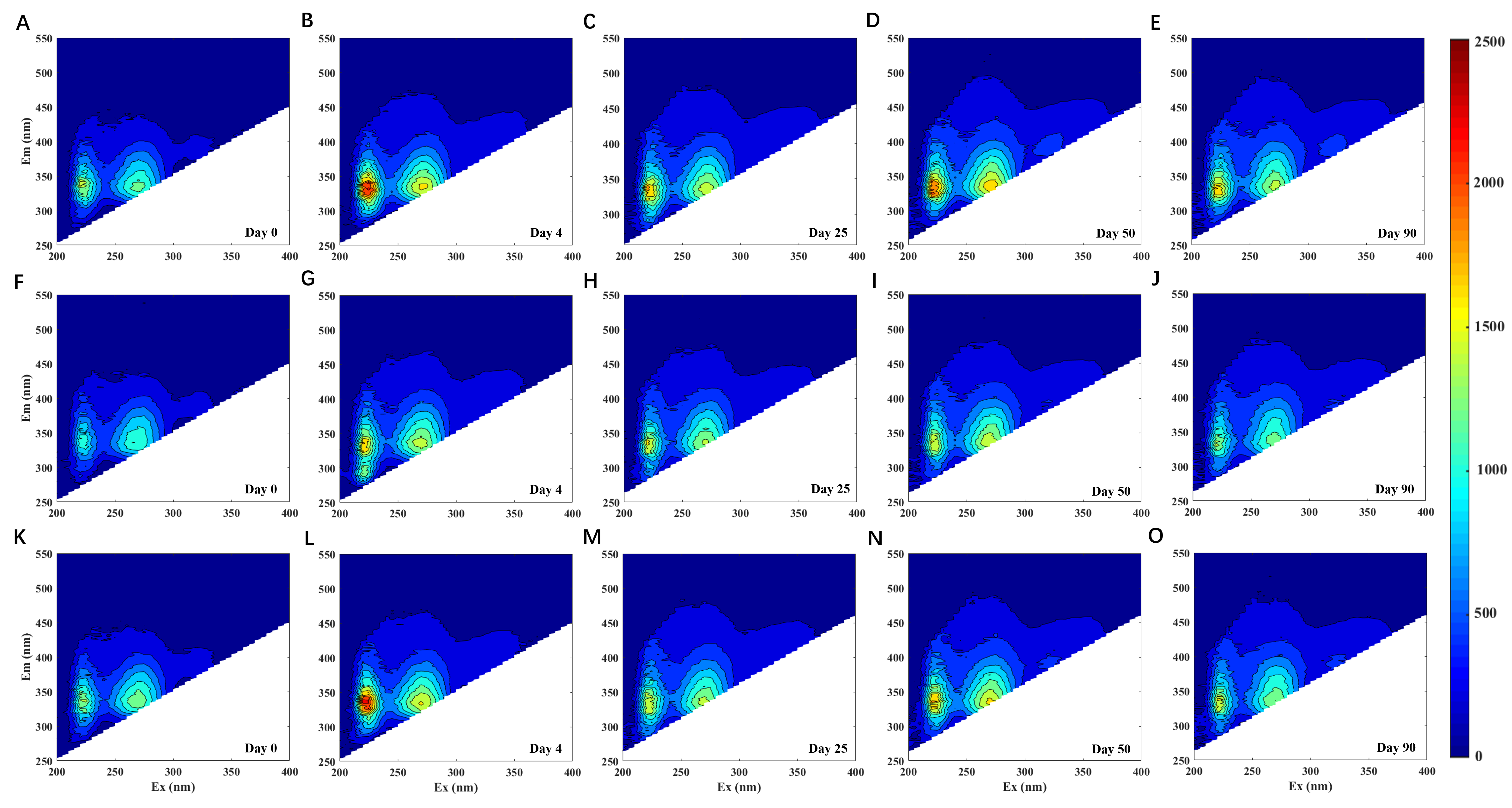


**Figure S2 Representative excitation-emission matrices (EEMs) of three culturable epiphytic bacteria transformed with *S. dohrnii*-derived DOM fluorescence at five time points (days 0, 4, 25, 50, and 90). A**-**E,** **F**-**J**, and **K**-**O** are represented as *Roseobacteria* sp. + DOM, *Marinobacter* sp. + DOM, and *Bacillus* sp. + DOM, respectively. The fluorescence intensity is given in arbitrary units (a.u.) with different scales.


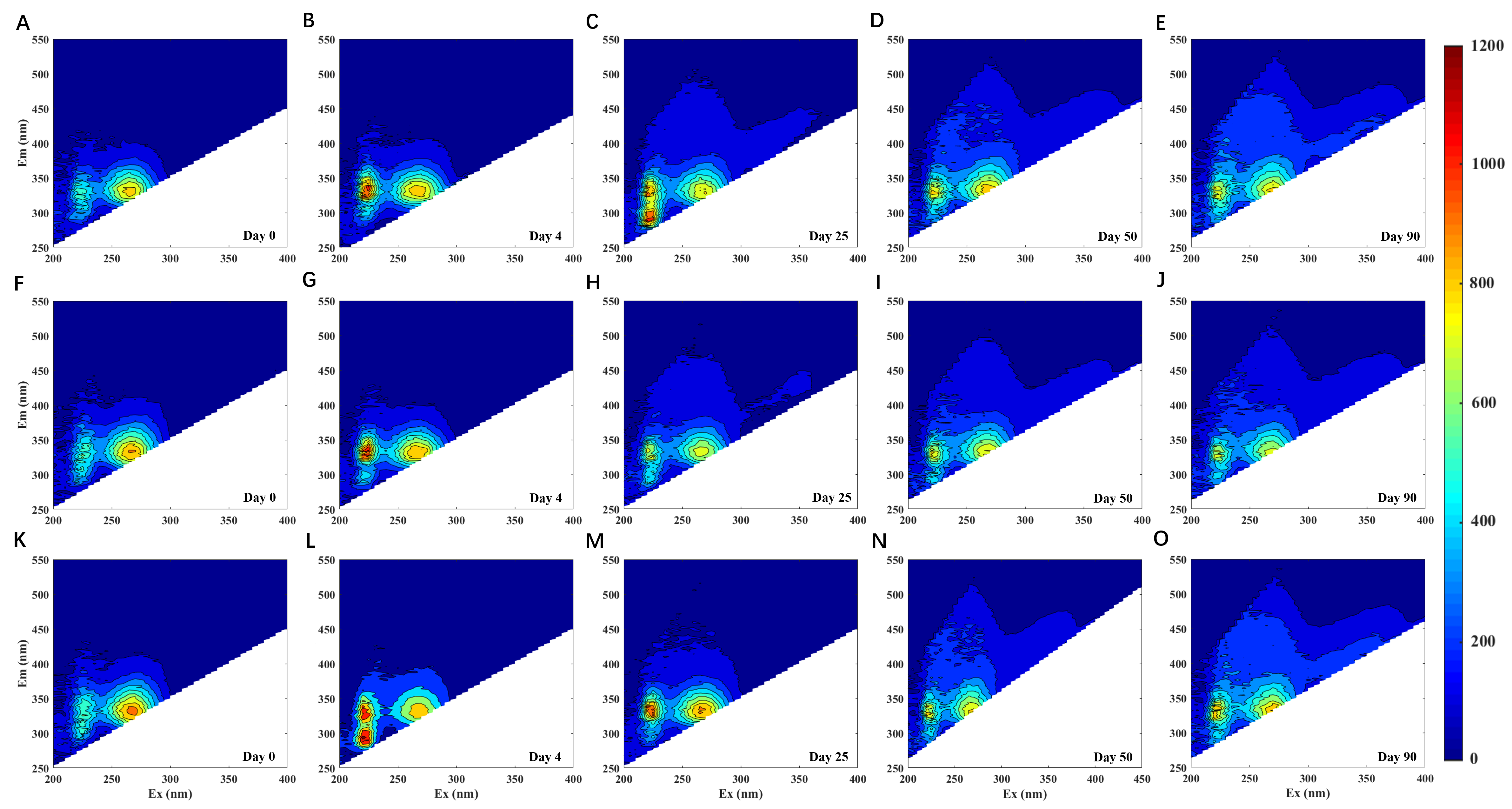


**Figure S3 Representative excitation-emission matrices (EEMs) of three culturable epiphytic bacteria transformed with *S. dohrnii*-derived LOM fluorescence at five time points (days 0, 4, 25, 50, and 90). A**-**E,** **F**-**J**, and **K**-**O** are represented as *Roseobacteria* sp + LOM., *Marinobacter* sp. + LOM, and *Bacillus* sp. + LOM, respectively. The fluorescence intensity is given in arbitrary units (a.u.) with different scales.
